# The exopolysaccharide of *Rhizobium* sp. YAS34 is not necessary for biofilm formation on *Arabidopsis thaliana* and *Brassica napus* roots but contributes to root colonization

**DOI:** 10.1111/j.1462-2920.2008.01650.x

**Published:** 2008-08

**Authors:** Catherine Santaella, Mathieu Schue, Odile Berge, Thierry Heulin, Wafa Achouak

**Affiliations:** 1 CEA, DSV, IBEB, SBVME, Laboratory Ecol Microb Rhizosphere and Environ Extrem (LEMiRE), Saint-Paul-lez-DuranceF-13108, France; 2 CNRS, UMR 6191, Saint-Paul-lez-DuranceF-13108, France; 3 Aix-Marseille Université, Saint-Paul-lez-DuranceF-13108, France

## Abstract

Microbial exopolysaccharides (EPSs) play key roles in plant–microbe interactions, such as biofilm formation on plant roots and legume nodulation by rhizobia. Here, we focused on the function of an EPS produced by *Rhizobium* sp. YAS34 in the colonization and biofilm formation on non-legume plant roots (*Arabidopsis thaliana* and *Brassica napus*). Using random transposon mutagenesis, we isolated an EPS-deficient mutant of strain YAS34 impaired in a glycosyltransferase gene (*gta*). Wild type and mutant strains were tagged with a plasmid-born GFP and, for the first time, the EPS produced by the wild-type strain was seen in the rhizosphere using selective carbohydrate probing with a fluorescent lectin and confocal laser-scanning microscopy. We show for the fist time that *Rhizobium* forms biofilms on roots of non-legumes, independently of the EPS synthesis. When produced by strain YAS34 wild type, EPS is targeted at specific parts of the plant root system. Nutrient fluctuations, root exudates and bacterial growth phase can account for such a production pattern. The EPS synthesis in *Rhizobium* sp. YAS34 is not essential for biofilm formation on roots, but is critical to colonization of the basal part of the root system and increasing the stability of root-adhering soil. Thus, in *Rhizobium* sp. YAS34 and non-legume interactions, microbial EPS is implicated in root–soil interface, root colonization, but not in biofilm formation.

## Introduction

Bacteria develop on plant roots as isolated cells, microcolonies and biofilms (Morris and Monier, 2003). Biofilms are assemblages of microbial cells that adhere to solid surfaces and are enmeshed in a self-produced extracellular matrix (Caldwell, 1995; Costerton *et al*., 1995). These structures provide cells with controlled living conditions that differ from the surrounding environment. The biofilm growth mode is a strategy for bacteria to invade their host in chronic versus acute infection (Furukawa *et al*., 2006).

In some plant–bacterial associations, exopolysaccharides (EPSs) are involved in adhesion of bacteria to roots (Michiels *et al*., 1991), root colonization (Matthysse *et al*., 2005) and are primary constituents of biofilms developed on plant roots (Bianciotto *et al*., 2001; Ramey *et al*., 2004; Fujishige *et al*., 2006).

In the root environment, i.e. the rhizosphere, bacterial EPSs contribute to soil aggregation by cementing particles together (Chenu, 1995). Inoculation of plants with EPS-producing rhizobacteria, such as *Pantoea agglomerans* and *Paenibacillus polymyxa* (Amellal *et al*., 1998; Bezzate *et al*., 2000), *Rhizobium* sp. YAS34 (Alami *et al*., 2000) and *Rhizobium* sp. KYGT207 (Kaci *et al*., 2005), modifies the aggregation of root-adhering soil and eventually improves plant growth.

The roles of EPS in the formation of biofilms on plant roots have principally been assessed using EPS-deficient or -overproducer mutants. However, in many studies, biofilms were seen on plant roots which were incubated in bacterial suspensions for short periods of time (Bianciotto *et al*., 2001; Bais *et al*., 2004; Walker *et al*., 2004; Matthysse *et al*., 2005; Timmusk *et al*., 2005; Fujishige *et al*., 2006). Moreover, the occurrence of EPS in bacterial root– biofilm matrix has never been seen by specific assays and on the roots of plants grown in soil and through long-standing plant–bacteria interactions. Yet, the development of root system and the soil matrix are environmental features that could influence the formation of biofilms on roots and the presence of EPS in the biofilm network.

In this study, we focused on the colonization of *Arabidopsis thaliana* and *Brassica napus* by an EPS-producing rhizobacterium *Rhizobium* sp. YAS34 (Alami *et al*., 2000), and the contribution of EPS synthesis to root colonization and biofilm formation using an EPS-deficient mutant of this strain. We have used confocal laser-scanning microscopy (CLSM) together with *gfp* tagging of bacterial cells and recognition-based selective probing of carbohydrates with lectins to target the EPS matrix. So far, lectins have never been used as reporters of carbohydrate and EPS in biofilms developed on plant roots and in the rhizosphere.

*Rhizobium* sp. YAS34 colonizes the whole-root system of *A. thaliana* and rapeseed plants as isolated cells, microcolonies and biofilms. This is the first evidence that *Rhizobium* forms biofilms on non-legume plant roots. We also show that at specific locations of the root system, the matrix of the *Rhizobium* sp. YAS34 biofilm is made of a bacteria-self-produced EPS. This bacterial EPS is not essential for biofilm formation on plant roots, but contributes to the colonization of specific zones in relation with nutrients availability.

## Results and discussion

### Selection of MS*Δ*GT, an EPS-deficient mutant of *Rhizobium* sp. YAS34 affected in a glycosyltransferase gene

**Fig. 1:**
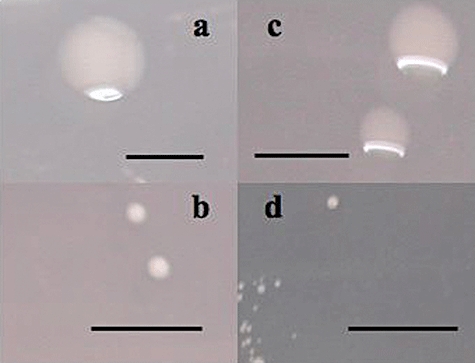
Phenotypes of *Rhizobium* sp. YAS34 grown 72 h at 30°C on RCV-agar medium supplemented with 2 g l^−1^ glucose. A. wt. B. Mutant MSΔGT. C. Mutant MSΔGT complemented with *gta* gene. D. Mutant MSΔGT complemented with an empty vector. Scale bar = 5 mm.

Using Tn*5*-based random mutagenesis in *Rhizobium* sp. YAS34 wild type (wt), the MSΔGT mutant strain was isolated as small non-mucoid colonies on agar plates containing RCV medium supplemented with glucose as sole carbon source (Fig. 1B). Sequencing of the chromosomal regions flanking the transposon showed that the interrupted gene encoded a putative glycosyltransferase named *gta* (Accession Number EU184019). We identified a putative complete gene including start and stop codons and a ribosome binding site. This gene was expressed in MSΔGT from the inducible P_*lac*_ promoter of the plasmid pBBR1-MCS3 in order to complement the mutation. This complemented mutant showed a mucoid phenotype (Fig. 1C) identical to that of the wt strain (Fig. 1A) while MSΔGT transformed with the empty plasmid pBBR1-MCS3 grew as non-mucoid colonies (Fig. 1D).

The EPS production in the parental, mutant and complemented strains was quantified by ethanol-precipitation of cultures in stationary phase and expressed as lyophilized weight per litre. *Rhizobium* sp. YAS34 wt produced up to 318 ± 32 mg l^−1^ of EPS whereas only a small amount of material (3 ± 3 mg l^−1^) could be isolated from cultures of the mutant using the same procedure. The complemented strain produced EPS (70 ± 7 mg l^−1^) and this production was enhanced to 135 ± 33 mg l^−1^ upon IPTG induction. Chemical structure of the isolated EPSs was confirmed by ^1^H NMR (data not shown), which was in agreement with the previously described EPS structure (Villain-Simonnet *et al*., 2000a).

Our results show that the *gta* gene was correctly predicted as encoding a glycosyltransferase in the YAS34 genome and could be expressed from pBBR1-MCS3 under the control of P_*lac*_. Using blast homology searches, the *gta* gene product composed of 402 aa was found to be similar to a glycosyltransferase from *Novosphingobium aromaticivorans* (45% identity at the protein level) and *Bradryrhizobium* (43%). In the CAZy database (http://www.cazy.org), such proteins are grouped in the GT4 family based on structural similarities, and mainly exhibit an alpha retention mechanism for sugar linkage. It is therefore likely that *gta* encodes a glucosyltransferase or a galactosyltransferase to add either glucose or galactose linked in alpha configuration in the monomeric structure of EPS from *Rhizobium* sp. YAS34 wt (B. Henrissat, pers. com.). We found that expression of this gene is essential for EPS production by this strain. Among the sequences of glycosyltransferases found most similar to the *gta* gene product in blast analysis, none have been shown to be functional *in vivo*. This result constitutes the first experimental evidence for *in vivo* activity of such glycosyltransferases.

### Biofilm formation by *Rhizobium* sp. YAS34 wt and its mutant MS*Δ*GT

We tested the ability of *Rhizobium* sp. YAS34 wt and its mutant MSΔGT to form biofilms in polypropylene tubes using a culture medium favourable for EPS synthesis with a crystal violet (CV) stain-based assay as described by O'Toole and Kolter (1998). First, YAS34 wt was compared with *Pseudomonas aeruginosa* (PAO1), which is a model organism for biofilm production. Levels of surface-attached cells of strain YAS34 were similar to those of PAO1 (data not shown). This result indicates that strain YAS34 wt is able to form biofilm when grown statically in polypropylene tubes. The EPS-deficient mutant, MSΔGT, showed identical levels of surface attachment to those seen in the parental strain (data not shown). Thus, EPS synthesis is not essential to that process, in our experimental conditions. However, the mutant could form biofilms at a slower rate than the parent strain and reached wt levels at the end of the experiment. Our results do not exclude that EPS could contribute to the kinetics of biofilm formation.

### Evidence for EPS synthesis in *Rhizobium* sp. YAS34 wt colonies growing on agar

**Fig. 2:**
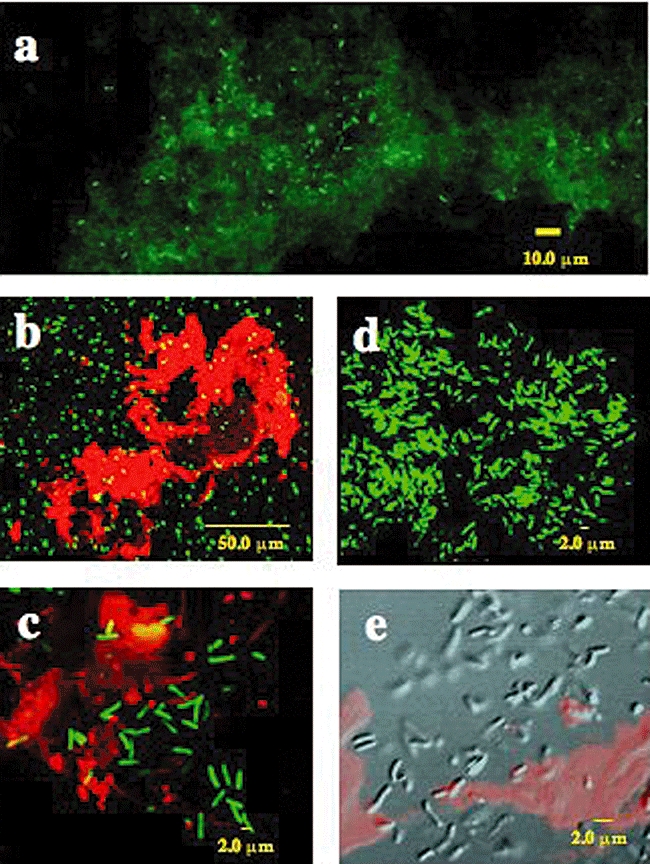
A. Localization of the EPS matrix in microcolonies of *Rhizobium* sp. YAS34 wt deposited on microscope glass slides, using the periodic acid-Schiff (PAS) reaction and carboxyfluorescein thiosemicarbazide reagent using CLSM. The EPS matrix was labelled by reaction of thiosemicarbazide with aldehyde functions generated after PAS reaction. Bacteria were visualized by labelling of glycoconjugates of the external membrane. Bacterial cells were entangled into a heterogeneous and cloudy EPS matrix alternating with dark zones. Projection of Z-sections (1 μm step) through 20.0 μm. Scale bar = 10 μm. B–E. Confocal laser-scanning micrographs of *Rhizobium* sp. YAS34 wt and mutant MSΔGT cells in biofilms grown on mineral medium supplemented with glucose as carbon source. Bacterial cells were localized by constitutive expression of GFP (in green) and EPS by binding with fluorescent Alexa660- or TR-ConA (in red). Overlays of GFP and red-fluorescent lectin emission channels (B–D) or red and transmission channels (E). B. Slime EPS of YAS34 wt was localized as discontinuous and large patches containing bacteria. Projection of Z-sections (1 μm step) through 50.0 μm. Scale bar = 50.0 μm. C. Microcolonies of YAS34 wt (in green) grown and stained with the fluorescent lectin (in red) inside an agar matrix. Note that all bacteria were not surrounded by EPS. Scale bar = 2.0 μm. D. Microcolonies of mutant MSΔGT stained with Alexa-660ConA. No staining was observed. Scale bar = 2.0 μm. E. Microcolonies of MSΔGT mutant complemented with a plasmid pBBR1-glycosyltransferase. The complementation restored labelling by Alexa660-ConA (in red). Bacterial cells were visualized by optical transmission. Scale bar = 2.0 μm.

The growth of bacteria on agar surfaces better mimics the conditions that bacteria experience in habitats such as soil, where water availability is influenced by the solute and matric potentials (Chang and Halverson, 2003). *Rhizobium* sp. YAS34 wt forms mucoid colonies on agar medium (Fig. 1A) in contrast to the EPS-deficient mutant that grows as small and rough colonies (Fig. 1B). To visualize the EPS matrix in these colonies by fluorescence microscopy, we modified the Thiery reaction (Thiery, 1967) generally used to localize carbohydrates in tissues, bacteria and soils (Erdos, 1986) by transmission electron microscopy. The substitution of thiocarbohydrazide and silver proteinate with a fluorescein thiosemicarbazide allowed fluorescent instead of electron-opaque staining of carbohydrates. This new method could allow fluorescent staining of an EPS for which no specific labelling is available. Linking of the fluorescent reagent with glycoconjugates of the cell membrane imaged the bacteria. *Rhizobium* sp. YAS34 wt mucoid colonies were stained with the modified Thiery reaction and examined in CLSM. Figure 2A shows an archetypical 3D structure of a biofilm with bacterial cells enmeshed in a glycoside-based matrix.

### ConcanavalinA specifically binds to the EPS produced by *Rhizobium* sp. YAS34 wt in biofilms

Based on the chemical structure of the EPS isolated from strain YAS34 wt (Villain-Simonnet *et al*., 2000a,b), the internal α-d-glucopyranosyle was targeted as a binding site for a lectin, concanavalinA (ConA) (Elgavish and Shaanan, 1997).

Turbidity measurements showed the specific interaction between this lectin and EPS from YAS34 wt. Addition of ConA to a suspension of EPS from strain YAS34 wt increased the turbidity by 75% (data not shown), which reflected binding of the lectin to the EPS chains. These aggregates dissociated when excess d-glucose, but not d-galactose, was added. Addition of excess d-glucose or d-galactose to the EPS suspension previous to that of the lectin, respectively, inhibited or permitted recognition.

Colonies of YAS34 wt, MSΔGT and its complemented mutant were grown on an agar-solidified mineral medium supplemented with glucose, suitable to EPS synthesis. Strains YAS34 wt and MSΔGT expressed a plasmid-born GFP. Cells of the complemented mutant were detected by direct optical transmission. Bacterial colonies were labelled with Alexa660-ConA (1 mg ml^−1^) and examined in CLSM (Fig. 2B–E). In *Rhizobium* sp. YAS34 wt and the complemented mutant, the bacteria were entangled within a three-dimensional network stained with ConA that revealed the presence of an EPS matrix (Fig. 2B, C and E). Yellow zones resulted from the superimposition of GFP-tagged bacteria and ConA-tagged matrix (Fig. 2B and C). Bacteria were also localized in areas that were not stained by ConA (Fig. 2B and C), showing that within the same population, some YAS34 wt cells did not produce EPS. Neither fluorescent staining nor EPS matrixes were detected when MSΔGT colonies were labelled with Alexa660-ConA and observed by CLSM (Fig. 2D).

Labelling of *Rhizobium* sp. YAS34 wt mucoid colonies with fluorescent ConA was inhibited in the presence of d-glucose, but not in the presence of d-galactose. Addition of d-glucose, but not d-galactose, to previously stained colonies with fluorescent ConA switched off the fluorescence (data not shown).

Altogether, macroscopic and microscopic data show that the matrix of *Rhizobium* sp. YAS34 wt biofilm contains an EPS, which is specifically recognized *in situ* by ConA.

### *Rhizobium* sp. YAS34 colonizes *A. thaliana* and *B. napus* roots independently of EPS production

The ability of EPS-producing and EPS-deficient strains of *Rhizobium* sp. YAS34 to colonize *A. thaliana* was investigated *in vitro* and in natural soil. Population sizes attached to the root system were determined by plate counts of serial dilutions of crushed roots. Per gram of dry root weight, 1.8 × 10^9^ ± 2.0 × 10^9^ cfu of *Rhizobium* sp. YAS34 wt (4.1 × 10^7^ ± 1.6 × 10^7^ cfu per root system) and 2.7 × 10^10^ ± 1.3 × 10^10^ cfu of mutant MSΔGT (6.0 × 10^7^ ± 2.9 × 10^7^ cfu per root system) were counted on *A. thaliana* ecotype Columbia roots of plantlets grown *in vitro* for 27 days. Similarly, 1.2 × 10^10^ ± 6 × 10^10^ cfu of YAS34 wt (4.2 × 10^8^ ± 1.9 × 10^8^ per root system) and 1.2 × 10^10^ ± 4 × 10^10^ cfu of MSΔGT (3.0 × 10^8^ ± 0.9 × 10^8^ cfu per root system) were counted on *B. napus* roots grown *in vitro* for 21 days. This corresponded to approximately 13 and 15 bacterial generations, respectively, on *A. thaliana* and *B. napus* roots. *In vitro*, the seeds were inoculated with a bacterial suspension. Therefore, the colonization occurred by adhesion of bacterial cells to young roots emerging from the seed. During growth, the root apex tends to diverge from the inoculation spot, driving a primary population that must utilize root exudates as sole carbon and energy source to persist on the root system. As bacteria do not grow on the medium used for the plant culture in the absence of plant roots (data not shown), the growth and development of bacterial cells are only dependent on root exudation. The fact that we observed similar numbers of EPS-producing and EPS-deficient bacteria colonizing roots indicates that EPS production is not necessary for root colonization *in vitro*.

In soil, 2.3 × 10^7^ ± 6.6 × 10^7^ cfu of strain YAS34 wt and 0.9 × 10^7^ ± 4.2 × 10^7^ cfu of mutant MSΔGT were counted per gram of dry root matter on *A. thaliana* ecotype Columbia plants grown for 90 days. Similarly, 1.1 × 10^8^ ± 0.6 × 10^8^ cfu of YAS34 wt and 2.9 × 10^7^ ± 1.3 × 10^7^ cfu of MSΔGT were counted per gram of dry root matter on *B. napus* grown for 33 days. The population of strains YAS34 wt and MSΔGT, respectively, accounted for 8% and 4% of the total culturable bacteria isolated from *A. thaliana* roots and for 5% and 1.3% of the total culturable bacteria isolated from *B. napus* roots. This result shows that *Rhizobium* sp. YAS34 wt and its mutant MSΔGT compete with other soil bacteria and colonize non-legume plants such as *A. thaliana* and *B. napus* in natural soil conditions, independently of EPS synthesis.

### *Rhizobium* sp. YAS34 forms biofilms on non-legume plant roots independently of EPS formation

**Fig. 5:**
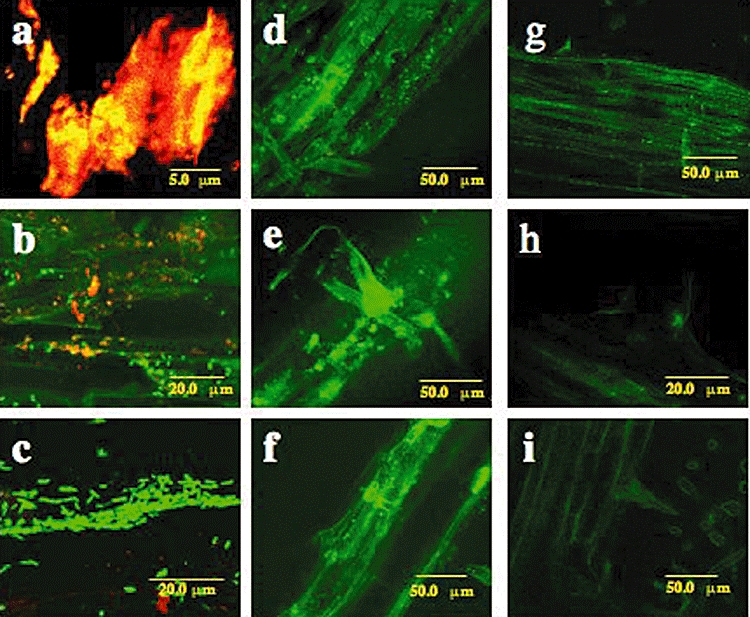
Colocalization of EPS and rhizobacteria on roots of rapeseed plantlets grown in soil using CLSM after Alexa660-ConA labelling. A–C. Overlays of green and red channels. Biofilm of *Rhizobium* sp. YAS34 wt developed (A) at the basal part of the root system. Bacteria (green or yellow) are wrapped round with a lectin-stained EPS matrix. Scale bar = 5.0 μm, (B) at the medium part of the root. Scale bar = 20.0 μm, (C) at the apical part of the root. Multilayers of cells are observed on the edge of the Z-projection of the root. Scale bar = 20.0 μm. D–F. Mutant MSΔGT colonizing rapeseed roots labelled with Alexa660-ConA. Scale bar = 50.0 μm. Isolated bacteria and patches of cells but no lectin-labelling were visualized (D) at the basis of the root system, (E) at the medium part of the root, (F) at the apical part of the root system. G–I. Axenic plantlet roots labelled with Alexa660-ConA as controls. On the green channel, the autofluorescence of root tissues allowed the visualization of plant cells. Non-inoculated plant roots did not bind the fluorescent lectin. (G) Basal part of the root. Scale bar = 50 μm. (H) At the median part of the root and on a lateral root starting. Scale bar = 20.0 μm. (I) At the apical part and root hairs of the root system. Scale bar = 50.0 μm.

**Fig. 3:**
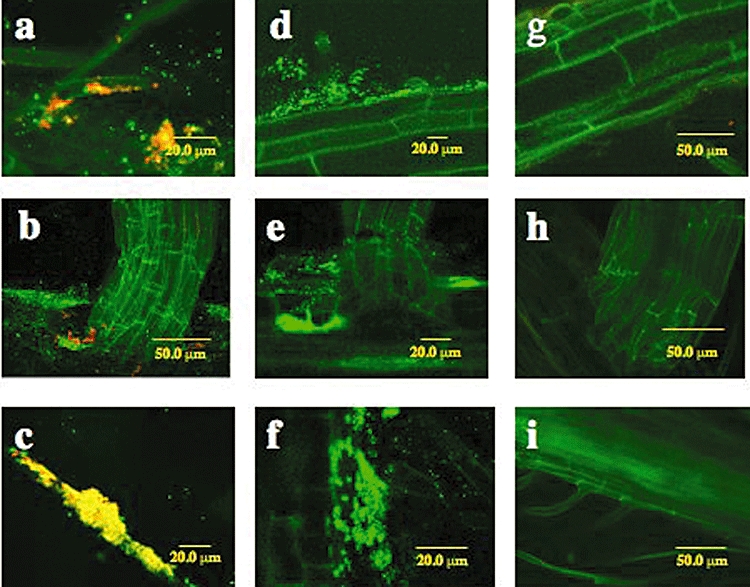
Biofilms of *Rhizobium* sp. YAS34 wt and mutant MSΔGT developed on the roots of *A. thaliana* plantlets grown *in vitro* using CLSM. A–C. Colocalization of *Rhizobium* sp. YAS34 wt expressing the GFP (in green) together with their EPS after Alexa660-ConA labelling (in red). Overlays of green and red channels. (A) At the medium part of the root. Z-stacks over 40.6 μm. Scale bar = 20 μm. (B) At the emergence of a lateral root. Z-projection over 17 μm. Scale bar = 50.0 μm. (C) On a root hair. Scale bar = 20.0 μm. D–F: Mutant MSΔGT expressing a plasmid-born GFP (in green) after labelling with Alexa660-ConA (in red). Overlays of green and red channels. No lectin binding was observed on the root of *A. thaliana* colonized by MSΔGT mutant. Scale bar is 20.0 μm. (D) At the basal part of the root, the three-dimensional organization of bacterial cells is obvious on the edge of the root projection. (E) Biofilm of mutant MSΔGT developed at the emergence of a lateral root. (F) Colonization of the apical part of *A. thaliana* root and on root hairs. G–I. Roots of axenic plantlet labelled with Alexa660-ConA as controls. No labelling with the lectin was visualized. Scale bar = 50.0 μm.

**Fig. 4:**
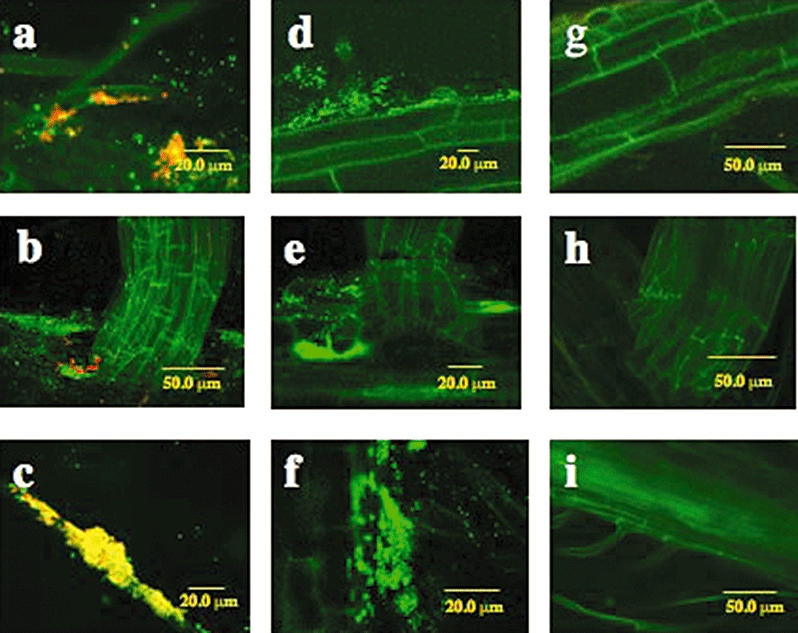
Evidence of *Rhizobium* sp. YAS34 wt and mutant MSΔGT biofilms on rapeseed plantlet roots grown *in vitro* using CLSM. A–C. Colocalization of YAS34 wt (in green) with its EPS after Alexa660-ConA labelling (in red). Overlays of green and red channels. (A) At basal part of the root. Projections of Z-sections (1 μm step) through 8 μm. Scale bar = 10.0 μm. In upper case, a detail of the biofilm structure, cells are entangled in a matrix labelled with the lectin. (B) At the emergence of a root hair. Projections of Z-sections over 20.0 μm (1 μm step). Scale bar = 20 μm. (C) At the apical part. Projections of Z-sections through 12 μm (1 μm step). Scale bar = 20.0 μm. D–F. Biofilms of mutant MSΔGT developed (D) at the basal part of the root system, (E) at the starting of a lateral root, (F) at the apical part of root. No EPS labelling was detected around the bacterial cells of mutant MSΔGT. G–I. Axenic plantlet roots labelled with Alexa660-ConA as controls. On the green channel, the autofluorescence of root tissues allowed the visualization of plant cells. On the red channel, no labelling with the lectin was observed. (G) Projections of Z-sections through 14 μm (1 μm step). Scale bar = 20.0 μm. (H) and (I) scale bar = 50.0 μm.

*Arabidopsis thaliana* and rapeseed seeds were inoculated with *Rhizobium* sp. YAS34 wt or its mutant MSΔGT expressing a plasmid-born GFP. *Arabidopsis thaliana* plants were grown *in vitro* and rapeseed *in vitro* and in non-sterilized field soil. As controls, axenic and non-inoculated *A. thaliana* and rapeseed plants were grown in the same conditions. At various times of plant growth, plant roots were labelled with Texas Red (TR)- or Alexa660-ConA and observed in CLSM. Figures 3 and 4 show representative images of the root systems of *A. thaliana* and *B. napus*, respectively, inoculated with *Rhizobium* sp. YAS34 wt (Figs. 3A–C and 4A–C) and mutant MSΔGT (Figs. 3D–F and 4D–F) or axenic (Figs. 3G–I and 4G–I) cultivated *in vitro*. Figure 5 shows typical illustrations of the roots of *B. napus* cultivated in natural soil conditions after inoculation with *Rhizobium* sp. YAS34 wt (Fig. 5A–C), mutant strain MSΔGT (Fig. 5D–F) or non-inoculated rapeseed plants (Fig. 5G–I).

*Rhizobium* sp. YAS34 wt and its mutant MSΔGT showed a similar pattern of colonization on roots of *A. thaliana* and those of rapeseed grown *in vitro*. The bacteria were found at the surface of plant epidermal cells and in interstitial zones along the whole-root systems (basal, medium and apical parts) as isolated cells, microcolonies and three-dimensional patches of cells that fulfil the basic definition of biofilms (Figs. 3A–F and 4A–F). Several authors already reported the colonization (Alami *et al*., 2000; Fujishige *et al*., 2006) or the nodulation (Trinick and Hadobas, 1995) of non-legumes by *Rhizobium* species. However, this is the first visualization of a biofilm from *Rhizobium* formed on non-legume plant roots.

YAS34 wt showed similar profiles of colonization on rapeseed roots grown *in vitro* or in soil (Figs. 4A–C and 5A–C). This suggests that *in vitro* culture systems are valuable devices for investigating biofilm formation by *Rhizobium* sp. YAS34 wt on plant roots. The same observation was found for the mutant MSΔGT except for the basal part of the root (Figs 4D–F and 5D–F). In this part of the root system, the mutant MSΔGT colonized the rapeseed root as patches of cells when the plants were grown *in vitro* while isolated cells were frequent on the root plants grown in soil.

A very important result of this study is that EPS production by *Rhizobium* sp. YAS34 wt was not critical for biofilm formation either on solid surfaces (polypropylene tubes) or on *Arabidopsis* and rapeseed roots. Attachment to root cells is the first step of bacterial root colonization and formation of biofilms on root surface. This primary step is mediated by various processes and molecules secreted by both partners of the plant–microbe interaction (Rodriguez-Navarro *et al*., 2007). For instance, outer cell-surface proteins, capsular polysaccharides or cellulose have been shown to be involved in attachment of, respectively, *Rahnella* (Achouak *et al*., 1998) *Pseudomonas*, *Azospirillum* and *Agrobacterium* (Rodriguez-Navarro *et al*., 2007). In the case of *Rhizobium* sp. YAS34, we show that the mechanism for root cell attachment does not require EPS synthesis and must involve other processes similar to those described above.

Biofilms developed by *Rhizobium* sp. YAS34 on plant roots growing *in vitro* or in soil were less extended than those formed on roots subcultured and dipped in bacterial cell suspensions (Bianciotto *et al*., 2001; Bais *et al*., 2004; Walker *et al*., 2004; Matthysse *et al*., 2005; Timmusk *et al*., 2005; Fujishige *et al*., 2006). Yet, the context of root–bacteria interaction in liquid culture systems differs from the environmental conditions that rhizobacteria face in the soil and in the rhizosphere. Actually, water availability together with nutrients drastically influence the dynamics and the development of biofilms (Chang and Halverson, 2003).

### ConcanavalinA binds to the EPS of *Rhizobium* sp. YAS34 produced *in planta*

For CLSM observations of roots, the tuning parameters were set as non-inoculated plant roots labelled with fluorescent derivatives of the lectin ConA (Figs 3D, 4D and 5D), and inoculated but unlabelled samples did not emit any fluorescence signal all along the root system (data not shown).

In our experiments, ConA did not bind to axenic roots (Figs 3G–I and 4G–I), root systems colonized by native soil rhizobacteria (Fig. 5G–I) or by mutant MSΔGT (Figs 3D–F and 4D–F). ConA-labelled areas were exclusively visualized on the root system of plants inoculated by *Rhizobium* sp. YAS34 wt. As controls, the lectin-labelled roots giving rise to some fluorescence of ConA-labelled zones were incubated in a solution of d-glucose. The competition of d-glucose for which ConA has a high affinity totally switched off the detection of stained areas (data not shown).

Altogether, these results demonstrate that ConA binds to the EPS produced by *Rhizobium* sp. YAS34 wt on plant roots.

### Exopolysaccharide localization *in planta*: EPS contributes to a better colonization of roots under natural conditions

On *A. thaliana* and rapeseed plant roots, the EPS production was dependent on plant age. On young plantlet roots of *A. thaliana* and rapeseeds (7 day old), inoculated with *Rhizobium* sp. YAS34 wt and grown *in vitro* or in soil, high densities of cells embedded in a translucent matrix were seen. However, labelling with ConA did not reveal the presence of an EPS matrix produced by *Rhizobium* sp. YAS34 wt (data not shown).

**Fig. 6:**
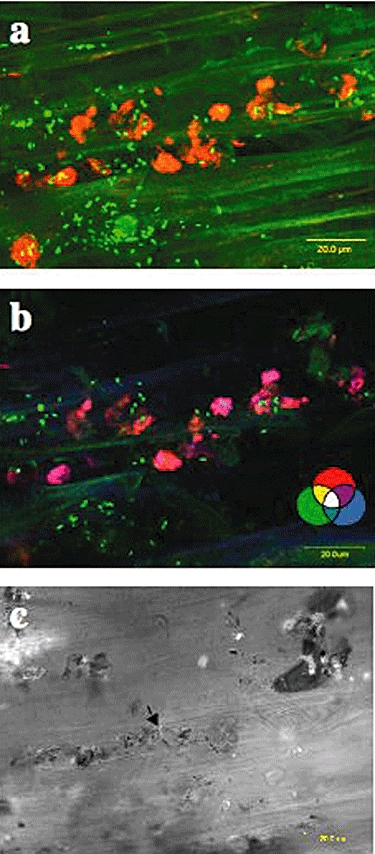
Development of *Rhizobium* sp. YAS34 wt biofilm on the root of rapeseed plant grown in soil using CLSM. Images represent the projection of Z-sections through 17 μm (1 μm step). A. Overlay of green and red channels emissions. Excitation at 488 and 645 nm allowed the localization of GFP-expressing bacteria and autofluorescence of root tissues in green and EPS bound to Alexa660-ConA (emission > 640 nm) in red. Some *Protozoa* were found grazing GFP-tagged bacteria. Scale bar = 20.0 μm. B. Overlay of the same zone, superimposing the signal obtained with excitation at 548 nm and emission between 585 and 640 nm. This allows imaging of the autofluorescent organic matter (shown in blue). Superimposition of artificial colours is given in overlapping circles. Scale bar = 20 μm. C. Observation of the same zone in transmission mode at *z* = 6 μm. Mineral particles (indicated by an arrow) were stacked to the root tissues and embedded into micro-aggregates. Scale bar = 20 μm.

Exopolysaccharides and biofilms were frequently seen on mature *A. thaliana* and rapeseed plant roots, respectively, grown for 15 or 30 days *in vitro* or in soil. In both culture conditions, biofilm formation on roots was repeatedly seen at the base of the root system. Figures 3A, 4A and 5A show three-dimensional EPS networks (surface area near 30 × 30 μm over a depth of 10 μm) that entangled GFP-expressing bacteria. At the base of the root system, microcolonies of *Rhizobium* sp. YAS34 wt were also found embedded in a three-dimensional EPS network (Fig. 6A) that enclosed autofluorescent soil organic matter (Fig. 6B). Transmission optical microscopy showed translucent mineral particles identified as crystal quartz that firmly adhered to these aggregates and resisted extensive washings of the root (Fig. 6C). Fluorescent lectins and CLSM were used to localize carbohydrates in various environments (Holloway, 1997; Neu and Lawrence, 1997; Langille and Weiner, 1998; Neu and Lawrence, 1999; Johnsen *et al*., 2000; Strathmann *et al*., 2002; Wigglesworth-Cooksey and Cooksey, 2005). However, this is the first report of the use of lectins to localize EPS in biofilms developed in the rhizosphere.

In biofilms, the possible roles for EPS-based matrix encompass protection against nutrient and water starvation, predation and chemical stress such as antimicrobial agents and reactive oxygen species (Wolfaardt *et al*., 1999). On plant roots, the composition, amount and spatial localization of root exudates and nutrient uptake are heterogeneous in time and in space (Martin, 1971; Walker *et al*., 2003). Environmental parameters could influence the formation of biofilms by *Rhizobium* sp. YAS34 at specific parts of the root.

Localization of *Rhizobium* sp. YAS34 wt and its EPS were similar on the roots of rapeseed grown either *in vitro* or in natural soil. However, the mutant MSΔGT showed a slightly reduced colonization of the basal part of the root system grown in soil compared with *in vitro*. This observation is supported by a minor but significant difference (*P* < 0.05) in bacterial population sizes determined on the roots of soil-grown rapeseed plants colonized by *Rhizobium* sp. YAS34 wt and its mutant MSΔGT. The basal part of the root system has higher nutrient influx rates than all other root zones (Liao *et al*., 2001; Rubio *et al*., 2001). Models for water uptake by plant roots show that water uptake sites are mostly regions near the base of the root system, close to the soil surface, and near the root tips (Roose and Fowler, 2004). Exopolysaccharide appears to be important for colonization of *Rhizobium* sp. YAS34 wt at the base of the root system, where an EPS-rich matrix could act as a trap for bacterial nutrition during water and solutes fluxes resulting from the sucking force of the plant.

Smaller biofilm-like structures of surface area 10 × 10 μm by a depth of 10 μm were observed throughout the whole-root system at the emergence of secondary roots and on root hairs (Figs 3B, C and 4B). The structure of bacterial aggregates on plant roots (Figs 4A and 6A) closely resembled that of microcolonies grown on an agar matrix where EPS producer bacteria coexisted with non-producer ones (Fig. 2B and C). The development of a lateral root generates fissures and releases galactosides that favour colonization by rhizobacteria (Bringhurst *et al*., 2001). Exopolysaccharide production at root cracking and on root hairs is therefore seen as expected in theses locations, but is not necessary to root colonization.

A high density of *Rhizobium* cells was often localized as microcolonies and monolayers on the apical part of *A. thaliana* and *B. napus* roots (Fig. 3C), and as multilayers at the apex (Vicre *et al*., 2005). However, EPS was rarely detected on these parts of the root system (Figs 4C and 5C). Carbon-rich material collected as exudates is predominantly released from root tip regions (McDougal and Rovira, 1970; Griffin, 1976; Darwent *et al*., 2003). However, in spite of high densities of bacterial cells in this part of the root, no EPS could be detected. Plant root tips release exudates such as glycosides (Haggquist *et al*., 1984; Lynch and Whipps, 1990). However, the hypothesis that glucose exudates could inhibit EPS interaction with the ConA lectin is rather inconsistent with the short half-lives of this compound in the rhizosphere (Coody *et al*., 1986; Kuzyakov and Jones, 2006). In liquid culture, *Rhizobium* sp. YAS34 wt starts to produce minute amounts of its EPS from the middle of exponential phase (data not shown). We therefore suggest that in short-term interactions and hence on the root tip, the physiological state of *Rhizobium* sp. YAS34 wt cells could be similar to an early exponential phase of microbial growth.

These results corroborate work from Foster and colleagues which reports that mucigel matrix is of microbial origin at the base and of plant origin on the younger parts of wheat root (Foster and Rovira, 1978; Foster, 1981).

### Exopolysaccharide synthesis by *Rhizobium* sp. YAS34 wt increases the stability of soil macro-aggregates

**Fig. 7:**
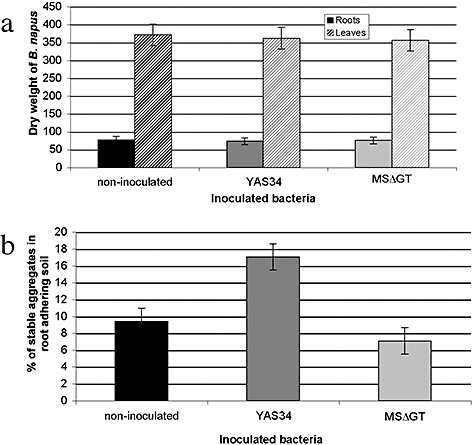
Effect of EPS production by *Rhizobium* sp. YAS34 wt in soil on (A) mass of dry plant of *B. napus* (in mg) and (B) soil structure modifications expressed as percentages of water-stable aggregates in root-adhering soil (*n* = 6 independent replicates).

*Brassica napus* seeds were inoculated by *Rhizobium* sp. YAS34 wt and mutant MSΔGT, and grown in soil for 33 days under controlled atmosphere. Inoculation of the two strains did not promote growth as non-inoculated control plants were found to have a similar mass of dry leaf and root (Fig. 7A). However, twice as much water-stable macro-aggregates were found in root-adhering soil inoculated with strain YAS34 wt compared with the EPS-deficient strain MSΔGT or in non-inoculated control treatment (Fig. 7B). This result strongly supports a role for rhizobial polysaccharides in modifying the soil structure by cementing soil particles around the roots of non-legumes (Alami *et al*., 2000). Together with the pictures of micro-aggregates stuck to the rhizoplane, these results show that the formation of biofilm by *Rhizobium* sp. YAS34 wt structures small root-adhering soil aggregates that resist more to water dispersion. These results definitely show a role of rhizobial polysaccharides in modifying the soil structure by cementing soil particles around the roots of non-legumes as hypothesized in Alami and colleagues (2000).

Soil structure determines the total volume of soil pores, their size distribution as well as their geometry and connectivity. The resulting properties of the soil and rhizosphere, such as aeration, resistance to root penetration, water reserve and therefore water and solute movements, are essential parameters that control plant growth. The stability of the soil structure is therefore one of the basic determinants of the soil and rhizosphere quality, if not of the ecosystem stability. Rhizobial EPS are essential in the successful symbiosis between rhizobia and legumes providing nitrogen to plants (Cheng and Walker, 1998; Fraysse *et al*., 2003). However, rhizobial populations persist owing to cells that do not participate in symbiosis but benefit from rhizodeposition for growth and survival (Bringhurst *et al*., 2001). In this study, we show that *Rhizobium* sp. YAS34 cells that produce EPS could be factors in promoting soil stability in the rhizosphere.

## Experimental procedures

### Bacterial strains and plasmids

*Rhizobium* sp. strains were grown at 30°C in 10-fold diluted tryptic soy broth (TSB/10) (Difco Laboratories, Detroit, USA) or in a RCV medium modified from Weaver and colleagues (1975) (MgSO_4_ 7H_2_O, 0.1 g l^−1^; CaCl_2_ 2H_2_O, 0.1 g l^−1^; FeSO_4_ 7H_2_O, 0.022 g l^−1^; EDTA 0.02 g l^−1^; ZnSO_4_ 7H_2_O, 0.43 mg l^−1^; MnSO_4_ H_2_O, 1.30 mg l^−1^; Na_2_MoO_4_ 2H_2_O, 0.75 mg l^−1^; H_3_BO_3_, 2.80 mg l^−1^; CuSO_4_ 5H_2_O, 26 μg l^−1^; CoSO_4_ 7H_2_O, 70 μg l^−1^; K_2_HPO_4_, 5.2 mM; KH_2_PO_4_, 4.4 mM, pH 6.8), supplemented with yeast extract (0.1 mg l^−1^) and glucose (2.0 g l^−1^) as carbon source to favour EPS synthesis. Nalidixic acid, kanamycin and tetracycline were, respectively, used at 50, 25 and 15 μg ml^−1^ for appropriate antibiotic selection of rhizobial strains.

*Rhizobium* sp. YAS34 wt (Alami *et al*., 2000) and its EPS-deficient mutant MSΔGT were labelled with GFP by triparental mating with *Escherichia coli* GM2163 (Marinus *et al*., 1983) carrying the pHC60 plasmid (Cheng and Walker, 1998) and *E. coli* LE392 carrying the helper plasmid pRK2013 (Figurski and Helinski, 1979) as described by Vicre and colleagues (2005). The constitutive expression of the GFP was stable for almost 3 months in soil, even in the absence of the antibiotic selection pressure (results not shown).

### Construction of an EPS-deficient mutant of *Rhizobium* sp. YAS34

Mutagenesis in *Rhizobium* sp. YAS34 wt was carried out by random insertion of a Tn*5* transposon from a non-replicative plasmid pRL1063a (Wolk *et al*., 1991). pRL1063a was transferred into YAS34 wt by triparental mating as described before and mutants by transposition were selected on TSB-agar plates containing kanamycin at 50 μg ml^−1^. Mutants were screened on RCV-agar plates supplemented with 2 g l^−1^ glucose to test for EPS production.

Genomic DNA from the YAS34 mutant was extracted, digested with ClaI (a restriction enzyme that does not cut within the Tn*5* sequence) (NEB), ligated with T4 ligase (Roche Diagnostics) and transferred into *E. coli* DH5α (Hanahan, 1983). As the transposon carries an origin of replication (p15A), only the plasmids containing Tn*5* and flanking regions from the YAS34 chromosome will replicate and maintain itself in *E. coli*. This plasmid was re-isolated and sequenced using unique primers OMP458 (5′-TACTAGATTCAATGCTATCAATTGAG-3′) and OMP459 (5′-AGGAGGTCACATGGAATATCAGAT-3′) directed outwards the Tn*5* ends (van den Broek *et al*., 2003).

### Complementation of MSΔGT strain

Based on the sequence obtained for DNA flanking regions of the transposon in MSΔGT, we identified a putative glycosyltransferase complete gene including start and stop codons and ribosome binding site. The gene sequence was submitted to GenBank under the Accession Number EU184019. Primers GlcTrXbaF (5′-CCCTATTCATCTAGAATGATAGGACCAGATatg-3′) and GlcTrSacR (5′-ATATGAGAGCTCTTCAtca agtaccagtctgaa-3′) were designed to amplify this complete gene (small uppercase letters indicate start and stop codons and underlined sequence show putative *rbs*). Upon PCR, an expected 1.2 kb fragment was obtained and cloned in pBBR-MCS3 (Kovach *et al*., 1995) downstream of a P_*lac*_ promoter using XbaI and SacI. The resulting plasmid, pBBR-*gta*, was transferred into MSΔGT by triparental mating as described before using 15 μg ml^−1^ tetracycline for selection and allowing an IPTG-inducible expression of the recombinant gene.

### Isolation of EPS from *Rhizobium* sp. YAS34 culture

The EPS-producing and EPS-deficient strains of *Rhizobium* sp. YAS34 were grown to stationary phase (OD_600nm_ = 0.2) in TSB/10 and bacterial cells were removed by centrifugation at 6000 *g* for 15 min at 4°C. Supernatants containing the soluble polysaccharide were collected and 2.5 vols of ice-cold ethanol were added. The EPS precipitated in these conditions and was harvested by 30 min centrifugation at 6000 *g* at 4°C. Supernatants were discarded and the EPS was re-suspended in a minimal water volume to be frozen at −20°C. After lyophilization, the amount of EPS was weighted and expressed as mg per litre of culture.

### Study of bacterial cell adhesion

To analyse the function of EPS in bacterial adhesion to solid surface and formation of biofilms, we used a CV staining-based protocol (O'Toole and Kolter, 1998) developed on polypropylene tubes. *Rhizobium* sp. YAS34 wt and mutant MSΔGT were grown statically to stationary phase in polypropylene tubes containing RCV medium supplemented with 2 g l^−1^ glucose. At an OD_600nm_ of 0.2, 1 ml of 1% CV solution was added and tubes were incubated for 20 min. Tubes were emptied and washed three times with ultra-pure water. Stained biofilms were re-suspended in 1 ml of absolute ethanol and the OD_590nm_ was measured in 96-well plates with 200 μl in each well. This OD value reflects the amount of surface-attached bacterial cells as described in O'Toole and Kolter (1998). Bacteria-free samples were stained using the same protocol and were taken as blanks.

### *Brassica napus* and *A. thaliana* growth conditions

Seeds of *B. napus* cv. Drakkar and *A. thaliana* (Columbia ecotype) were surface-sterilized and sown in square plates (12 × 12 cm or 25 × 25 cm) containing sterile half-strength Hoagland nutrient solution (Arnon and Hoagland, 1940) solidified with Phytagel (Sigma, 7 g l^−1^) (Achouak *et al*., 2004). Each seed was inoculated with 5 μl of a *Rhizobium* sp. YAS34 suspension (about 10^6^ cfu ml^−1^) grown to late exponential phase, washed twice with sterile KCl (0.85 g l^−1^) and then re-suspended in sterile ultra-pure water. On control plants, 5 μl of sterile ultra-pure water was added to the seeds. The plates were sealed with Micropore tape (3M, St Paul, MN, USA) and incubated vertically at 21°C with 16 h of light (100 mmol m^−2^ s^−1^) and 19°C for 8 h light off. Plantlets were harvested after 7, 15 and 21 days of culture for *B. napus* and 7, 15, 21 and 28 days for *A. thaliana.*

For plant culture in natural soil, we used the upper 30 cm of the soil profile of a typical Eutric cambisol (FAO-UNESCO classification) of INRA (Achouak *et al*., 2004). The last crop before sampling was wheat. Topsoil contained clay (17.5%), silt (53.0%) and sand (29.5%), organic carbon (1.35%) and nitrogen (0.12%). Water pH was 7.85 ± 0.05. Soil was air-dried, passed through 2 mm mesh sieve, hydrated to 13% of humidity and stored at room temperature for at least 10 days before bacterial inoculations and plant sowing. Soil was inoculated with *Rhizobium* sp. YAS34 cultures (wt and MSΔGT mutant, 10^5^ cfu g^−1^ of dry soil, final humidity 18%) and poured onto pots with a density of 1.2 (dry weight per volume of pot). Seeds of *B. napus* cv. Drakkar were surface-sterilized and sown in pots. Plantlets were grown in chambers with controlled atmosphere for 15, 21 and 33 days in the conditions described above. Seeds of *A. thaliana* ecotype Columbia were surfaced-sterilized, inoculated with 5 × 10^3^ cfu of the two YAS34 strains and germinated for 8 days on half-strength Hoagland medium solidified with 0.7% Phytagel. Plantlets were transferred to pre-inoculated soil and grown in chambers for 90 days under controlled atmosphere, 22°C with 8 h of light (120 mmol m^−2^ s^−1^) and 19°C for 16 h light off, 60–70% relative humidity.

### Enumeration of bacterial populations developed on roots

Root-adhering bacteria were counted on the whole-root system. Three to five root systems per treatment were washed in 100 ml of ultra-pure water for roots of plantlets grown in natural soil and crushed. The bacterial populations were estimated by plating serial dilutions in KCl 0.85 g l^−1^ on TSB/10 agar containing tetracycline (15 μg ml^−1^) and counting of the cfu after 3 day incubation at 30°C.

### Interaction between EPS of *Rhizobium* sp. YAS34 wt and ConA

Stock solutions of ConA (Sigma and Molecular Probes, 1 mg ml^−1^) were prepared in 0.2 μm filtered phosphate buffer saline (10 mM, pH 6.8), supplemented with MnCl_2_ and CaCl_2_ (0.1 mM each, Sigma), and stored at 4°C.

Interaction between the lectin and the EPS was evidenced by monitoring the timecourse increase of turbidity at 600 nm of 1 ml solution of EPS (1 mg ml^−1^) upon increasing lectin concentrations (up to 1 mg ml^−1^) in water or in PBS. Then, a solution of d-glucose or d-galactose (Sigma, 25 mg ml^−1^) was added and the optical density was time-monitored.

### Imaging the EPS produced by *Rhizobium* sp. YAS34 wt with ConA

Colonies of *Rhizobium* sp. YAS34 expressing a plasmid-born GFP were scrapped from the agar surface and deposited on a slide. One hundred microlitres of tetramethylrhodamine isothiocyanate (TRITC)- or Texas Red (TR-ConA) (Molecular probes) solution (1 mg ml^−1^ and 0.1 mg ml^−1^) in PBS containing CaCl_2_ and MnCl_2_ (0.1 mM each) were added and the slide was stored 15 min in the dark. The excess solution was then discarded and the slide-rinsed in 40 ml of PBS for 15 min. In some experiments, slides were incubated in glucose (25 mg ml^−1^) before or after the addition of ConA.

Alternatively, *Rhizobium* sp. YAS34 colonies grown inside agar-solidified medium were directly labelled by addition of TR-ConA solution (1 mg ml^−1^) and interaction during 12 h at 4°C. The plate was rinsed twice and incubated overnight with water at 4°C, then directly observed under the microscope.

Roots of plantlets grown *in vitro* were labelled on the plates by pouring the lectin solution (0.1 mg ml^−1^ in PBS, MnCl_2_, CaCl_2_) all over the roots. Alternatively, a whole plant was removed from the plate and incubated into 10 ml of lectin solution for 30 min. For plants grown in soil, the plants were harvested, gently hand-shacked and rapidly immerged first into 40 ml of water to remove non-adhering soil, then in 40 ml of TRITC- or Alexa660-ConA (0.2 mg ml^−1^ in PBS, CaCl_2_, MnCl_2_ buffer) for 1 h. In some cases, bovine serum albumin (grade II, Sigma, 0.2 g l^−1^) was added in the staining solution to saturate unspecific sites such as clays and reduce unspecific binding of ConA.

Excess labelling solution was discarded or the plant was removed from the labelling solution. The roots were washed twice with sterile water, and incubated for 1 h in water or PBS buffer. Plant roots were observed directly on the growth-medium surface with coverslip or mounted between a slide and a coverslip, with citifluor (Interchim) or water.

As controls, non-inoculated plantlets were labelled with the procedure described above and inoculated plantlets were labelled in the same way except the addition of the fluorescent lectin.

Plantlet roots for which numerous zones of bacteria and EPS had been colocalized were incubated in the presence of a glucose solution (20 mg l^−1^) for 15 min and rinsed with water. The plantlets were observed again in the same scanning conditions.

### Modified Thiery reaction for labelling of carbohydrates

This procedure was adapted from the Thiery reaction (Thiery, 1967), used to detect glycopolymers having 1,2-diol by transmission electron microscopy. The thiocarbohydrazide-silver and proteinate reagents were replaced by a fluorescent thiosemicarbazide (Sigma, 0.02% in sodium acetate solution, pH 5.5) that could be visualized in CLSM.

### Microscopic observations

An Olympus CLSM system equipped with krypton-argon lasers (488, 568, 647 nm lines) and oil objectives was used for microscopic observations. Lectin tagged with FITC, TRITC-, TR- or Alexa660 were excited with 488 (green channel), 568 and 647 nm wavelengths (red channel) respectively. 488 and 568 nm scanning were acquired separately. Emissions were observed with appropriate filters (510–560 nm on green channel, 585–640 nm or > 660 nm on red channel). Samples were examined in the fluorescence and in the transmission mode. Axenic plants labelled with the fluorescent lectin and *Rhizobium* sp. YAS34 unlabelled inoculated plantlets were used as control samples. In each experiment, these control samples were observed by CLSM in order to determine acquisition parameters leading to an absence of fluorescent signal (i.e. dark images) on the channel used for the detection of the fluorescent lectin. Observation of unlabelled plantlets gives information on the autofluorescence level of the sample. Observation of axenic plantlets labelled with the fluorescent lectin reports on the selectivity of the probe. At least six plantlets (three plantlets from two independent experiments) of each treatment (inoculated or non-inoculated) were observed along the whole-root material, in order to achieve a valid and representative analysis of the samples.

### Measuring soil aggregate stability

Sterilized seeds of *B. napus* Drakkar were sown in soil either inoculated with *Rhizobium* sp. YAS34 wt or MSΔGT mutant or non-inoculated plants were grown for 33 days (*n* = 6 replicates) in controlled atmosphere. Roots were harvested with root-adhering soil at a relative soil humidity of 16% and left to dry before they were passed onto a 250 μm sieve. Small aggregates of adhering soil were recovered and weighted. Sieves containing roots and large aggregates of root-adhering soil were then deepened into water and agitated for several minutes. Stable macro-aggregates remained on the top of the sieve whereas water-disrupted aggregates passed through and were collected in water. Each fraction (washed roots, stable macro-aggregates and water-disrupted macro-aggregates) were collected, dried and weighted. Percentages of dry stable macro-aggregate mass in total dry root-adhering soil mass were determined for each plant and treatment and submitted to statistical analysis of variance using statgraphics*Plus*.

## References

[b1] Achouak W, Pages JM, De Mot R, Molle G, Heulin T (1998). A major outer membrane protein of *Rahnella aquatilis* functions as a porin and root adhesin. J Bacteriol.

[b2] Achouak W, Conrod S, Cohen V, Heulin T (2004). Phenotypic variation of *Pseudomonas brassicacearum* as a plant root-colonization strategy. Mol Plant Microbe Interact.

[b3] Alami Y, Achouak W, Marol C, Heulin T (2000). Rhizosphere soil aggregation and plant growth promotion of sunflowers by an exopolysaccharide-producing *Rhizobium* sp. strain isolated from sunflower roots. Appl Environ Microbiol.

[b4] Amellal N, Burtin G, Bartoli F, Heulin T (1998). Colonization of wheat roots by an exopolysaccharide-producing *Pantoea agglomerans* strain and its effect on rhizosphere soil aggregation. Appl Environ Microbiol.

[b5] Arnon DI, Hoagland DR (1940). Crop production in artificial culture solutionsand in soils withspecial reference to factorsinfluencing yields and absorption of inorganic nutrient. Soil Sci.

[b6] Bais HP, Fall R, Vivanco JM (2004). Biocontrol of *Bacillus subtilis* against infection of *Arabidopsis* roots by *Pseudomonas syringae* is facilitated by biofilm formation and surfactin production. Plant Physiol.

[b7] Bezzate S, Aymerich S, Chambert R, Czarnes S, Berge O, Heulin T (2000). Disruption of the *Paenibacillus polymyxa* levansucrase gene impairs its ability to aggregate soil in the wheat rhizosphere. Environ Microbiol.

[b8] Bianciotto V, Andreotti S, Balestrini R, Bonfante P, Perotto S (2001). Mucoid mutants of the biocontrol strain *Pseudomonas fluorescens* CHA0 show increased ability in biofilm formation on mycorrhizal and nonmycorrhizal carrot roots. Mol Plant Microbe Interact.

[b9] Bringhurst RM, Cardon ZG, Gage DJ (2001). Galactosides in the rhizosphere: utilization by *Sinorhizobium meliloti and* development of a biosensor. Proc Natl Acad Sci USA.

[b10] van den Broek D, Chin A, Woeng TFC, Eijkemans K, Mulders IHM, Bloemberg GV, Lugtenberg BJJ (2003). Biocontrol traits of *Pseudomonas* spp. are regulated by phase variation. Mol Plant Microbe Interact.

[b11] Caldwell DE, Lappin-Scott HM, Costerton JW (1995). Cultivation and study of biofilm communities. Microbial Biofilms.

[b12] Chang W-S, Halverson LJ (2003). Reduced water availability influences the dynamics, development, and ultrastructural properties of *Pseudomonas putida* biofilms. J Bacteriol.

[b13] Cheng HP, Walker GC (1998). Succinoglycan is required for initiation and elongation of infection threads during nodulation of alfalfa by *Rhizobium meliloti*. J Bacteriol.

[b14] Chenu C, Huang PM, Berthelin J, Bollag JM, McGill WB, Page AL (1995). Extracellular polysaccharides: an interface between microorganisms and soil constituents. Environmental Impact of Soil Components Interactions.

[b15] Coody PN, Sommers LE, Nelson DW (1986). Kinetics of glucose-uptake by soil-microorganisms. Soil Biology Biochem.

[b16] Costerton JW, Lewandowski Z, Caldwell DE, Korber DR, Lappin-Scott HM (1995). Microbial biofilms. Annu Rev Microbiol.

[b17] Darwent MJ, Darwent MJ, Paterson E, Paterson E, McDonald AJS, McDonald AJ (2003). Biosensor reporting of root exudation from *Hordeum vulgare* in relation to shoot nitrate concentration. J Exp Bot.

[b18] Elgavish S, Shaanan B (1997). Lectin–carbohydrate interactions: different folds, common recognition principles. Trends Biochem Sci.

[b19] Erdos GW, Aldrich HC, Todd WJ (1986). Localisation of carbohydrates-containing molecules. Ultrastructure Techniques for Microorganisms..

[b20] Figurski DH, Helinski DR (1979). Replication of an origin-containing derivative of plasmid RK2 dependent on a plasmid function provided in trans. Proc Natl Acad Sci USA.

[b21] Foster RC (1981). Polysaccharides in soil fabrics. Science.

[b22] Foster RC, Rovira AD, Loutit MW, Miles JAR (1978). The rhizosphere. The Ultrastructure of the Rhizosphere of Trifolium Subterraneaum L.Microbial Ecology.

[b23] Fraysse N, Fraysse N, Couderc F, Couderc F, Poinsot V, Poinsot V (2003). Surface polysaccharide involvement in establishing the *Rhizobium*-legume symbiosis. Eur J Biochem.

[b24] Fujishige NA, Kapadia NN, De Hoff PL, Hirsch AM (2006). Investigations of *Rhizobium* biofilm formation. FEMS Microbiol Ecol.

[b25] Furukawa S, Kuchma SL, O'Toole GA (2006). Keeping their options open: acute versus persistent infections. J Bacteriol.

[b26] Griffin GJ (1976). Nature and Quantity of sloughed organic-matter produced by roots of axenic peanut plants. Soil Biol Biochem.

[b27] Haggquist ML, Svenningsson H, Olsson S, Sundin P, Odham G, Liljenberg C (1984). Long-term culturing of plants with aseptic roots – determination of rape root exudates. Plant Cell Environ.

[b28] Hanahan D (1983). Studies on transformation of *Escherichia coli* with plasmids. J Mol Biol.

[b29] Holloway CF (1997). Development of a scanning confocal laser microscopic technique to examine the structure and composition of marine snow. Limnol Oceanogr.

[b30] Johnsen AR, Hausner M, Schnell A, Wuertz S (2000). Evaluation of fluorescently labeled lectins for noninvasive localization of extracellular polymeric substances in *Sphingomonas* biofilms. Appl Environ Microbiol.

[b31] Kaci Y, Heyraud A, Barakat M, Heulin T (2005). Isolation and identification of an EPS-producing *Rhizobium* strain from arid soil (Algeria): characterization of its EPS and the effect of inoculation on wheat rhizosphere soil structure. Res Microbiol.

[b32] Kovach ME, Elzer PH, Hill DS, Robertson GT, Farris MA, Roop RM, Peterson KM (1995). Four new derivatives of the broad-host-range cloning vector pBBR1MCS, carrying different antibiotic-resistance cassettes. Gene.

[b33] Kuzyakov Y, Jones DL (2006). Glucose uptake by maize roots and its transformation in the rhizosphere. Soil Biology Biochem.

[b34] Langille SE, Weiner RM (1998). Spatial and temporal deposition of *Hyphomonas* strain VP-6 capsules involved in biofilm formation. Appl Environ Microbiol.

[b35] Liao H, Rubio G, Yan X, Cao A, Brown KM, Lynch JP (2001). Effect of phosphorus availability on basal root shallowness in common bean. Plant Soil.

[b36] Lynch JM, Whipps JM (1990). Substrate flow in the rhizosphere. Plant Soil.

[b37] Marinus MG, Carraway M, Frey AZ, Brown L, Arraj JA (1983). Insertion mutations in the dam gene of *Escherichia coli* K-12. Mol Gen Genet.

[b38] Martin JK (1971). Influence of plant species and plant age on rhizosphere microflora. Aust J Biol Sci.

[b39] Matthysse AG, Marry M, Krall L, Kaye M, Ramey BE, Fuqua C, White AR (2005). The effect of cellulose overproduction on binding and biofilm formation on roots by *Agrobacterium tumefaciens*. Mol Plant Microbe Interact.

[b40] McDougal B, Rovira AD (1970). Sites of Exudation of C-14-Labelled Compounds from Wheat Roots. New Phytol.

[b41] Michiels KW, Croes CL, Vanderleyden J (1991). Two different modes of attachment of *Azospirillum brasilense* sp7 to wheat roots. J Gen Microbiol.

[b42] Morris CE, Monier J-M (2003). The ecological significance of biofilm formation by plant-associated bacteria. Annu Rev Phytopathol.

[b43] Neu TR, Lawrence JR (1997). Development and structure of microbial biofilms in river water studied by confocal laser scanning microscopy. FEMS Microbiol Ecol.

[b44] Neu TR, Lawrence JR (1999). Lectin-binding analysis in biofilm systems. Methods Enzymol.

[b45] O'Toole GA, Kolter R (1998). Initiation of biofilm formation in *Pseudomonas fluorescens* WCS365 proceeds via multiple, convergent signalling pathways: a genetic analysis. Mol Microbiol.

[b46] Ramey BE, Koutsoudis M, von Bodman SB, Fuqua C (2004). Biofilm formation in plant–microbe associations. Curr Opin Microbiol.

[b47] Rodriguez-Navarro DN, Rodriguez-Navarro DN, Dardanelli MS, Dardanelli MS, Ruiz-Sainz JE, Ruiz-Sainz JE (2007). Attachment of bacteria to the roots of higher plants. FEMS Microbiol Lett.

[b48] Roose T, Fowler AC (2004). A model for water uptake by plant roots. J Theor Biol.

[b49] Rubio G, Walk T, Ge ZY, Yan XL, Liao H, Lynch JP (2001). Root gravitropism and below-ground competition among neighbouring plants: a modelling approach. Ann Bot.

[b50] Strathmann M, Wingender J, Flemming H-C (2002). Application of fluorescently labelled lectins for the visualization and biochemical characterization of polysaccharides in biofilms of *Pseudomonas aeruginosa*. J Microbiol Methods.

[b51] Thiery JP (1967). Demonstration of polysaccharides in thin sections by electron microscopy. J Microsc (Oxford).

[b52] Timmusk S, Grantcharova N, Wagner EGH (2005). *Paenibacillus polymyxa* invades plant roots and forms biofilms. Appl Environ Microbiol.

[b53] Trinick MJ, Hadobas PA (1995). Formation of nodular structures on the non-legumes *Brassica-napus*, *Brassica-Campestris*, *Brassica-Juncea* and *Arabidopsis-thaliana* with *Bradyrhizobium* and *Rhizobium* isolated from *Parasponia spp* or legumes grown in tropical soils. Plant Soil.

[b54] Vicre M, Santaella C, Blanchet S, Gateau A, Driouich A (2005). Root border-like cells of *Arabidopsis*. Microscopical characterization and role in the interaction with rhizobacteria. Plant Physiol.

[b55] Villain-Simonnet A, Milas M, Rinaudo M (2000a). A new bacterial exopolysaccharide (YAS34). II. Influence of thermal treatments on the conformation and structure. Relation with gelation ability. Int J Biol Macromol.

[b56] Villain-Simonnet A, Milas M, Rinaudo M (2000b). A new bacterial exopolysaccharide (YAS34). I. Characterization of the conformations and conformational transition. Int J Biol Macromol.

[b57] Walker TS, Bais HP, Grotewold E, Vivanco JM (2003). Root exudation and rhizosphere biology. Plant Physiol.

[b58] Walker TS, Bais HP, Deziel E, Schweizer HP, Rahme LG, Fall R, Vivanco JM (2004). *Pseudomonas aeruginosa*–plant root interactions. Pathogenicity, biofilm formation, and root exudation. Plant Physiol.

[b59] Weaver PK, Wall JD, Guest H (1975). Characterization of *Rhodopseudomonas capsulata*. Arch Microbiol.

[b60] Wigglesworth-Cooksey B, Cooksey KE (2005). Use of fluorophore-conjugated lectins to study cell–cell interactions in model marine biofilms. Appl Environ Microbiol.

[b61] Wolfaardt GM, Lawrence JR, Darren RK, Wingender J, Neu T, Flemming HC (1999). Function of EPS. Microbial Extracellular Polymeric Substances: Characterization, Structure and Function..

[b62] Wolk CP, Cai Y, Panoff JM (1991). Use of a transposon with luciferase as a reporter to identify environmentally responsive genes in a cyanobacterium. Proc Natl Acad Sci USA.

